# 351 Investigation of a Corynebacterium amycolatum Sternal Surgical Site Infection Cluster in Cardiac Surgery

**DOI:** 10.1017/ash.2026.10690

**Published:** 2026-06-23

**Authors:** JaHyun Kang

**Affiliations:** 1 Seoul National University College of Nursing

## Abstract

**Background:** During outbreaks of emerging infectious diseases, healthcare personnel (HCP) safety depends not only on PPE availability but also on correct donning and doffing. In real-world isolation care, inconsistent guidance, product variability, and workflow constraints contribute to self-contamination risk. Human-factors–driven PPE ensembles were developed and their performance was evaluated through a series of standardized, simulation-based randomized crossover studies. **Methods:** Three randomized crossover simulation studies were performed among HCP to evaluate PPE performance. The first study compared existing Level C/D PPE ensembles with prototype PPE ensembles during simulations (n=40). Participants performed two donning–simulation–doffing sequences with randomized PPE order, and self-contamination was assessed using fluorescent markers and ultraviolet light. Based on findings from the first study, the PPE ensembles were iteratively refined and re-evaluated in a second randomized crossover simulation focused on post-refinement performance among returning participants (n=29). A third randomized crossover simulation assessed the effect of structured donning/doffing education and brief coached practice when using the refined PPE ensembles compared with existing PPE among PPE-na HCP (n=20). Outcomes included objective self-contamination events, perceived protection, usability (donning, working, doffing), overall satisfaction, confidence, and intention to use. **Results:** In the initial randomized crossover simulation (n=40), self-contamination after doffing was observed in 42.5% of participants using existing PPE and 52.5% using the prototype PPE ensembles, despite higher perceived convenience with these ensembles (mean scores, 10-point scale: working 7.5 vs 5.9, doffing 6.6 vs 5.5). Following iterative refinement, the second simulation (n=29) demonstrated improved performance of the developed PPE ensembles, with self-contamination reduced to 37.9% compared with the initial prototype evaluation, and no severe contamination events observed. Usability ratings further improved (donning 8.0 vs 7.1, working 8.1 vs 7.5, doffing 7.8 vs 6.6), and intention to use was high (8.9/10). In the third randomized crossover simulation among PPE-na HCP (n=20), structured education and coached practice reduced self-contamination from 50% with existing PPE to 35% with the developed PPE ensembles and substantially improved usability (donning 8.6 vs 6.1, working 9.0 vs 6.4, doffing 8.4 vs 5.2) and confidence. **Conclusions:** Across three randomized crossover simulation studies, human-factors–driven PPE ensembles demonstrated improvements in usability and reductions in self-contamination. Importantly, early identification of increased self-contamination risk during prototype evaluation enabled design optimization, resulting in safer performance. Standardized simulation-based evaluation can identify early risks, guide PPE optimization, and support safer adoption of improved PPE ensembles in high-risk isolation care settings.

